# Reviewing the Benefits of Grazing/Browsing Semiarid Rangeland Feed Resources and the Transference of Bioactivity and Pro-Healthy Properties to Goat Milk and Cheese: Obesity, Insulin Resistance, Inflammation and Hepatic Steatosis Prevention

**DOI:** 10.3390/ani11102942

**Published:** 2021-10-12

**Authors:** Claudia Delgadillo-Puga, Mario Cuchillo-Hilario

**Affiliations:** 1 Departamento de Nutrición Animal Dr. Fernando Pérez-Gil Romo, Instituto Nacional de Ciencias Médicas y Nutrición Salvador Zubirán (INCMNSZ), Tlalpan 14080, Ciudad de México, México; 2 Departamento de Ciencias Pecuarias, Facultad de Estudios Superiores de Cuautitlán, Universidad Nacional Autónoma de México, Cuatitlán Izcalli 54714, Estado de México, México

**Keywords:** bioactive compounds, antioxidant activity, n-3, fatty acids, phenols, healthy foods, phytochemicals

## Abstract

**Simple Summary:**

The aim of this study was to review the benefits of producing milk and cheese under grazing/browsing systems on rangelands in terms of bioactivity and the health benefits of their consumption in these animals. Firstly, we looked over at the forages consumed by goats on the rangelands and at the plant’s bioactive compounds. Additionally, goat milk and cheese coming from (1) grazing animals, (2) animals managed indoors, and from (3) animals managed indoors, but supplemented with rich plant bioactive compounds, were examined. In the end, milk was analyzed to modulate the negative effects of high-fat diet in mice. The results revealed that grazing/browsing practices are superior to indoor feeding to promote the transference of bioactive compounds from vegetation to animal tissues, and finally to animal products. Grazing/browsing management represents a better option than indoor feeding to enhance the bioactivity of milk and cheese. Supplementation with rich-bioactive compound forages increased total polyphenol, hydroxycinnamic acids, and flavonoid concentrations in both products. The consumption of goat milk prevents obesity, insulin resistance, inflammation, and hepatic steatosis in mice.

**Abstract:**

The rangeland is an ecological resource that provides multiple benefits for environment and agriculture. Grazing/browsing on rangelands is a useful and inexpensive means to produce food derived from animal products. The aim of this study was to review the benefits of producing milk and cheese under this system in terms of bioactivity and the health benefits of their consumption in model animals. To conduct this review, we particularly considered the experiments that our research group carried out along the last fifteen years at the Instituto Nacional de Ciencias Médicas y Nutrición Salvador Zubirán in Mexico. Firstly, we examined the forages consumed by goats on the rangelands in terms of plant bioactive compound occurrence and their concentration. Further, goat milk and cheese coming from (1) grazing animals, (2) animals managed indoors, and from (3) animals managed indoor supplemented with rich plant bioactive compounds, were analyzed. Milk was discussed to modulate the negative effects of high-fat diets in mice. Forages consumed by goats on the rangelands showed a close correlation between antioxidant activity assessed by the DPPH^+^ radical with total flavonoid and total polyphenol contents (TPC). Milk concentration of PUFA, MUFA, and n-3 fatty acids from grazing goats (4.7%, 25.2%, and 0.94% of FAME) was higher than milk from goats fed indoor diets (ID). Similar results were shown in cheese. TPC was higher in cheese manufactured with milk from grazing goats (300 mg of GAE/kg of cheese) when compared to cheese from milk goats fed ID (60 mg of GAE/of cheese). *Acacia* pods are a semiarid rangeland feed resource that transfers pro-healthy activity, inhibited in vitro lipid peroxidation (inhibition of TBARS formation) and diminished the damage induced by reactive oxygen species (ROS). Additionally, in vivo assessment revealed that *Acacia* species increased free radical scavenging (DPPH), oxygen radical absorbance capacity, and anti-inflammatory activity. The results highlight that grazing/browsing practices are superior to indoor feeding in order to promote the transference of bioactive compounds from vegetation to animal tissue, and finally to animal products. Grazing management represents a better option than indoor feeding to enhance bioactivity of milk and cheese. Supplementation with rich-bioactive compound forages increased total polyphenol, hydroxycinnamic acids, and flavonoid concentrations in milk and cheese. The consumption of goat milk prevents obesity, insulin resistance, inflammation, and hepatic steatosis while on a high-fat diet induced obesity in mice.

## 1. Introduction

Grazing/browsing is a useful and inexpensive tool to improve the presence of bioactive compounds and by this means increase the bioactivity of milk and cheese [1]. This type of feeding is a sustainable alternative for ruminants’ production, if available natural resources and animals’ needs are harmonized [2,3]. This means of production is crucial to support food security and to increase household income of smallholders around the world [4]. Highly diverse pastures concentrate a wide range of plant bioactive compounds (PBC) that are better than monoculture or poor-species grasslands [3,5]. Likewise, grazing/browsing practices have proved to be superior over indoor feeding to promote the transference of bioactive compounds from vegetation to animal metabolism and to animal products [6,7,8]. These effects have been confirmed in different animal species and in distinct food animal products [5,9,10,11].

Though most goat production systems in developing countries face pitfalls such as poor forage quality, water scarcity, or inconstant feed supply throughout the seasons; such animal farming has the potential to produce dairy products with pro-healthy properties [2,12]. Despite the fact that many variables modify the content of bioactive compounds of milk and cheese, it is well accepted that animal feeding is the factor that has the greatest impact [13,14]. In addition, indoor feeding has shown benefits in terms of bioactivity if the animal diet includes substantial bioactive ingredients that surpass the animal’s biological metabolism and reaches the milk and cheese components [15,16,17,18]. Anti-inflammatory, anticarcinogenic, and cardioprotective effects of phytonutrients [19,20] are the most common desirable effects that might be found in milk and cheese [21,22]. This sheds light into why goat milk and cheese have claimed benefits for human nutrition and health [23,24].

In the recent years remarkable advances have been made to understand the impacts of sustainable ruminant farming systems and the benefits to human nutrition and health [25,26,27]. Different approaches have been taken to identify the compounds involved in such claimed benefits and the likely pathways followed by such compounds to exhibit pro-healthy benefits in food deliveries. In line with this, the bioactive compounds that our research group has reported in goat milk and cheese included poly-unsaturated fatty acids, flavonoids, catechin, hydroxycinnamic acids, phenols, and terpenes, among others. The bioactive compounds ingested by animals coming from plant species and the potential benefits of the compounds later found in dairy deliveries should be fully identified to better understand the metabolic mechanisms to increase their occurrence and their concentration in order to exert such favorable biological functions [12]. Further, this information should have practical application to provide appropriate recommendations regarding the likely effects of their intake by humans. To reach these objectives, it is necessary to investigate goat browsed/grazed vegetation.

To elucidate the potential sources of plant bioactive compounds [28] and to analyze the botanical components to confirm the origin of these phytochemicals [29], analyses were made. Some species or theirs parts exhibited special properties beyond their nutritional value, e.g., *Acacia farnesiana* [15,30]. Some other species performed well as a rich source of anti-inflammatory agents, while also protecting against oxidative-induced damage in biological models e.g., *A. farnesiana* and *A. schaffneri* [31]. Our recent findings showed that bioactivity of dairy products is enhanced when goats are under browsing/grazing management or supplemented with *A. farnesiana* pods meal [15]. As well, goat milk ingestion can support antioxidant activity of biological systems and even modulate chronic disease while regulating inflammatory indicators to prevent obesity and related maladies [32].

To our knowledge, there is a gap in the literature regarding the traceability of bioactive compounds consumed by animals on rangelands, their occurrence in goat milk and cheese and the likely benefits to curb maladies as obesity and related disorders in animal models. In the present review, goat milk [8,15] and cheese [6,7,8,13] were revisited on their bioactive compound content. In addition, in vitro and in vivo assays were considered to have the evidence of the effects of such compounds [30,31,32]. To conduct this review, we took into account particularly the experiments that our research group carried out throughout the last fifteen years at the Instituto Nacional de Ciencias Médicas y Nutrición Salvador Zubirán (INCMNSZ) in Mexico. The initial works of our research group dealing with botanical screening, nutritive value and sustainability of rangelands started in the late 90s and the early 00s [28,33] and has continued until recent times [34]. In addition, the grazing/browsing effect on quality of animal products has been a topic of our interest. In this review, we summarized the most meaningful findings of grazing/browsing semiarid rangeland feed resources to increase bioactivity and pro-healthy properties to goat cheese and milk.

We proved that goat milk intake prevents chronical illness in animal models. In this publication, “rangeland” was defined according to Allen et al. [35], as the “land on which indigenous vegetation is predominantly grass, grass-like plants, forbs, or shrubs that are grazed, or have the potential to be grazed, and which is used as a natural ecosystem for the production of grazing livestock”. Likewise, secondary metabolite was defined according to Martirosyan et al. [36] as “the molecules in food, usually in small amounts, that act synergistically to benefit health”.

## 2. Materials and Methods

### 2.1. Experiment 1

The rangeland used for this experiment was located in Queretaro, Mexico (20°35′Northern latitude, 100°18′Western longitude: 1950 m.a.s.l.). The area has a dry, semiarid climate with 460 mm of average precipitation per year. The vegetation included forbs, leguminous trees and *Opuntia* species (Table 1). Sampling of grazed/browsed species was performed by hand simulating the mouthful of the goat. The sampling included leaves, stems, fruit or a combination of them, in line with the natural ingestion patterns of goats on the rangelands. Rangeland vegetation species were collected and dried on three different days and sampling was done twice on every observational day (n = 6). Analysis of antioxidant activity on vegetation samples was done according to Sharma and Bhat [37]; while total phenol content was determined according to Folin–Ciocalteu colorimetric method described by Taga et al. [38]. Goats had an average weight of 50 ± 5 kg and a lactation period of 150 days. Two animal groups were set up with French Alpine goats. (1) The grazing group fed exclusively local vegetation species available in the rangeland area. (2) Indoor feeding management consisted of grain concentrate (rolled corn 55%, wheat bran 17%, barley 15%, soybean 9.3%, and vitamins and minerals 3.7%) supplemented with Lucerne hay. Both animal groups were managed with overnight confinement. The grazing group did not receive any additional feed supply. Goats were milked daily at 7:00 a.m. Milk from each replicate group of five animals each (two replicate groups per treatment were selected) was collected during five consecutive days. Further, soft artisan cheese was manufactured and analyzed [8]. Total milk fat was determined according to Folch et al. [39]. Further, lipids were dissolved in hexane and sodium hydroxide methanol solutions for saponification. Later, transesterification of milk fat to methyl esters were done following the recommendations of the official method 696.33, AOAC [40]. Finally, fatty acid methyl esters (FAME) were quantified by gas chromatography [8].

### 2.2. Experiment 2

For the second experiment, French Alpine goats (50 ± 5 kg) were selected and a lactation period of 150 days were allocated into three groups, as follows: (1) grazing/browsing goats; (2) conventional diet (CD); and (3) conventional diet supplemented with 30% of *Acacia farnesiana* pods meal (AF). Goats were housed in herds of ten animals each. Grazing animals were allowed to graze/browse during 8 h/d on 14 ha of shrubby rangeland after milking with overnight confinement. Animals fed conventional diet or supplemented conventional diet were kept in full confinement along the experiment. Feedstuffs for indoor feeding was harvested (Lucerne hay) and prepared (grain concentrate) once for the complete experimental period and stored separately. The proportion used was 60/40 (Lucerne hay/grain concentrate) for indoor feeding; while 42/28/30 (Lucerne hay/grain concentrate/*A. farnesiana* pods meal) proportion was used in the supplemented diet. Milk samples of each animal group were collected separately in seven consecutive days following the adaptation period of 12 days [15]. Fatty acid profile determination was performed the first experiment. Total phenolic content was determined by the Folin–Ciocalteu colorimetric [41] with some modifications described by Puga et al. [15]. Antioxidant activity of milk was carried out according to Koren et al. [42]. Flavonoids and phenolic acids were determined by HPLC [15].

### 2.3. Experiment 3

French Alpine with Saanen and Toggenburg crossbred-goats (120 goats) were allowed to pasture 14 ha of shrub land per day. The average weight of the adult females was 55 ± 5 kg, and they were in mid-lactation. The management of the goats included daily grazing on the rangeland after milking with overnight confinement. Sixty goats were kept indoors in full confinement during the study. Milk from each group of goats was pooled and artisan cheese was manufactured [6]. Fatty acid profile determination was performed as Experiments 1 and 2. Total phenolic content was determined by the Folin–Ciocalteu colorimetric test [41] with some modifications described by Puga et al. [15]. Antioxidant activity of milk was carried out according to Koren et al. [42]. Mono and sesquiterpenes volatile compounds were analyzed by Purge and Trap Dynamic Head Space technique and separated by gas chromatography. Further, terpenes were identified by mass spectrometry [6].

### 2.4. Experiment 4

Two groups of 40 French Alpine goats that weighed 50 ± 5 kg was arranged. All animals had 70 to 80 milking days. Grazing group grazed/pastured 8 h/d on 14 ha of natural rangeland with overnight confinement supplemented with 200 g of concentrate grain (18% CP) in the morning and 300 g Lucerne hay in the evening. The indoor group was kept in full confinement during the study, fed 1 kg (18% of CP and 2.5 Mcal/kg) of grain concentrate supplemented with 1.5 kg Lucerne hay/day. The goats were milked once daily at 7:00 h along five days. Soft artisan cheese was manufactured and analyzed [13]. Fatty acid profile determination was performed for Experiments 1, 2, and 3.

### 2.5. Experiment 5

This experiment used the same animals and conditions of Experiment 1, but instead of evaluating goat milk, this experiment was designed to assess soft goat cheese [8]. In addition, complementary parameters such as antioxidant activity and bioactive compounds were analyzed to better characterize goat cheese [7]. Determination of fatty acid profile and total phenolic content was determined as Experiment 2 [15]. Antioxidant activity was calculated with the methodology of Hatano et al. [43], while flavonoids and hydroxycinnamic acids were measured by HPLC [7].

### 2.6. Experiment 6

In this experiment, two assays were performed to determine the antioxidant activity and protection against oxidative induced damage of crude extracts of *Acacia farnesiana* and *A. schaffneri* pods. It is important to remember that both plant species are rich sources of phytochemicals and are important components of goat diet in browsing/grazing rangeland management. Firstly, the protective effect of crude extracts of *Acacia* pods were challenged against H_2_O_2_ using pig kidney LLC-PK1 cells in an in vitro assay. Secondly, gerbils (*Meriones unguiculatus*) were employed (in vivo assay) to observe the effect of crude extract of *Acacia* pods on plasma antioxidant capacity [31]. Free radical scavenging capacity of *A. schaffneri* and *A. farnesiana* was carried out using the decoloration reduction of ABTS•^+^ assay from Re et al. [44], while DPPH^+^ assay was performed according to Von Gadow et al. [45], and liposomes measured according to Tsuda et al. [46]. To evaluate antioxidant activity in pig kidney cells LLC–PK1 line cells were employed as Hernández–Fonseca et al. recommended [47]. DPPH^+^ scavenging capacity in gerbil plasma was determined according to Koren et al. [42], while oxygen radical absorbance capacity (ORAC) assays were based on the method described by Huang et al. [48].

### 2.7. Experiment 7

To follow up with the characterization of goat feeds from semiarid rangelands as valuable resources of bioactive compound source; five organic extracts (chloroformic, hexanic, ketonic, methanolic, methanolic:aqueous, and one aqueous extract) of *A. farnesiana* pods were tested in vitro and in vivo. All extracts were challenged to investigate their antioxidant activity and protection against oxidative-induced damage as well as their capacity to curb the inflammation process and to downregulate the pro-inflammatory mediators [30]. Total phenolic content was determined by the Folin–Ciocalteu colorimetric method described by Singleton et al. [49]. To evaluate the quantitative antioxidant activity, DPPH• (2,2-diphenyl-1-picrylhydrazyl) reagent was employed according to Koren et al. [42]. For the in vivo model, eight groups of mice with six animals each were employed. Ear edema (weight, and thickness ear) essay in CD-1 mice induced with 12-o-tetradecanoylphorbol acetate (TPA), oxidative enzyme myeloperoxidase assay (MPO), histological analysis of ear, immunohistochemistry, quantitation of IL-1β, IL-6, and tumor necrosis factor alpha (TNF-α) tests were done according to Del–Ángel et al. [50] and Delgadillo et al. [51].

### 2.8. Experiment 8

In this experiment, the effect of (1) milk from goats fed a conventional diet, (2) milk from conventional diet supplemented by 30% *A. farnesiana* pods and (3) milk from grazing goats on metabolic alterations in mice fed a high fat diet was evaluated. Male C57BL/6 mice were housed in micro-isolator cages. These animals were randomly assigned into five groups (n = 6) receiving the following diets: (1) control; (2) high-fat (HF); (3) HF + dry milk from goats fed a conventional diet (HFCD); (4) HF + dry milk from goats fed on grazing (HFG); and (5) HF + dry milk from goats fed a conventional diet supplemented with 30% of *A. farnesiana* pods. Body weight, body composition (percentage of fat and lean mass), energy expenditure measurement, intraperitoneal glucose, insulin tolerance test, histological analysis of liver, pancreas, white and brown adipose tissue, mitochondria abundance, and lipid content in skeletal muscle and liver were evaluated. Likewise, immunoblotting and immunohistochemistry in BAT of UCP-1, and TNF-α quantitation in adipose tissue were evaluated. Further details of the employed methodology can be found in our previous paper [32].

### 2.9. Statistical Analyses

Total polyphenol content and quantitative radical scavenging activity of plant samples were analysed by ANOVA (*p* = 0.05) using SAS (SAS Institute Inc, Cary, NC, USA) [52]. The days of collection were treated as repeated measurements. For each plant portion (complete fruits, leaves and stems; cladodes for *Opuntia* species) we used the nonparametric statistic of K independent samples. The Kruskal–Wallis test was used to establish differences among plant portions. Further, the Mann–Whitney U signed ranks test for related pairs of portions was used to identify such differences [53]. In vivo effects on bioactivity and healthy properties against induced obesity were evaluated by one-way ANOVA, followed by Tukey multiple comparison post hoc test using GraphPad Prism 7.0 (GraphPad Software, San Diego, CA, USA). The differences were considered statistically significant at *p* < 0.05.

## 3. Results

### 3.1. Antioxidant Activity and Total Polyphenol Content of Vegetation Species Browsed/Grazed by Goats on Semiarid Rangelands

**Figure 1:**
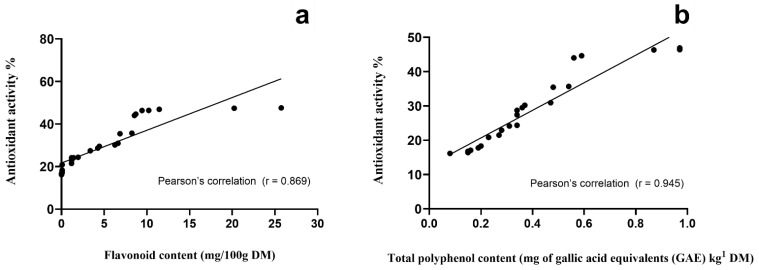
Pearson’s correlations of flavonoid (**a**) and total polyphenol (**b**) content versus antioxidant activity of vegetation species browsed/grazed by goats on semiarid rangelands.

For the analysis of antioxidant activity of rangeland vegetation, complete plants yielded the best anti-free-radical performance, followed by fruits (including pods), stems, and leaves, respectively (Table 1). Our results revealed a close Pearson’s correlation of the antioxidant activity assessed by the DPPH+ radical with total flavonoid (r = 0.869; Figure 1a) and total polyphenol content (TPC; r = 0.945; Figure 1b). In addition, fruits from rangeland vegetation species had the highest mean value for TPC, while cladodes had the lowest (*p* < 0.001). In line with this outcome, the same association was observed when stems and cladodes were contrasted (*p* < 0.001). However, when individual species were analyzed, *A. farnesiana* pods showed the largest TPC (38,170 mg of gallic acid equivalents/kg DM) value, followed by *A. schaffneri* (2730 mg of gallic acid equivalents/kg MD). This remarkable TPC associated with the antioxidant activity parameter were the reasons to utilize both feed resources in the following experiments to better know the extent of their utilization in goat feeding and the likely changes in bioactivity and pro-health properties of milk and cheese.

### 3.2. Bioactive Compounds in Goas Milk and Cheese

#### 3.2.1. Fatty Acids in Goat’s Milk and Cheese

In Experiment 1 (Table 2), the largest concentration of polyunsaturated fatty acids (PUFA) and monounsaturated fatty acids (MUFA) was found in milk coming from grazing goats (4.7% and 25.2% of fatty acid methyl esters—FAME), followed by milk from goats fed conventional diets (3.4% and 19.9% of FAME). In the same line, n-3 fatty acids concentration in milk from grazing goats was superior to indoor diets (0.94% vs. 0.72%, respectively). However, the maximum saturated fatty acids (SFA) mean value was obtained in milk from goats fed conventional diets (70.3% of FAME) in comparison to grazing management (64.4%). In contrast to Experiment 1; in Experiment 2 we found that milk from indoor depicted with the top value of PUFA (5.6%), MUFA (31.6%) and n-3 fatty acids (0.96%) content. On the other hand, grazing treatment and indoor group supplemented with 30% of *A. farnesiana* (AF) pods increased the content of SFA of goat milk (69.7% and 69.4% of FAME, respectively) in relation to conventional diet, conventional diet plus 10% of AF and conventional diet plus 20% of AF (62.7%, 62.8%, and 62.8% of FAME, respectively). Moreover, conjugated linoleic acid (CLA) was found in higher proportion in grazing treatment (0.29%; versus the rest of the treatments which average 0.22% of FAME).

In line with Experiment 1; Experiment 3 showed that grazing management increases PUFA and MUFA (6.1% and 23.5%) fatty acids share of cheese in relation to indoor feeding (5.2% and 23.8%). Likewise, the SFA was found lower for grazing system (68.9%) and higher for cheese from indoor diet (69.4%). For the total concentration of n-3 fatty acids, no differences were detected. In Experiment 4, the results indicated that PUFA were higher in cheese made with goat milk from indoor diet (5.1%) in relation to grazing feeding (3.9%). For the total concentration of n-3 series we observed no differences. However, the total concentration of n-6 fatty acids in cheese from goats fed indoor diet was higher when compared to the cheese elaborated with milk from grazing goats. Therefore, at the end, the n-3:n-6 ratio was more advantageous for grazing system (0.36 vs. 0.26). In the case of Experiment 5, grazing management decreased PUFA content (4.8%) in relation to indoor management (5.4%). However, MUFA value was superior in cheese made with milk from grazing goats (25.3%) over the cheese coming from goats fed a conventional diet (23.9%).

#### 3.2.2. Phenolic Content, Flavonoids, Terpenes, Hydroxycinnamic Acids, and Antioxidant Activity in Goat’s Milk and Cheese

Apart from the fatty acid profile, the inclusion of AF pods in the goat’s diets improved the total phenolic content in the milk (Experiment 2) significantly. The highest concentration of total phenols was found in the indoor diet supplemented with AF (305.5 mg of gallic acid equivalents—GAE/L of milk), while indoor diet, i.e., indoor diet without supplementation was the lowest in this respect (159.4 mg of GAE/L of milk). The grazing system also increased this parameter, but at a lesser extent than indoor diet plus AF supplementation (231.6 mg of GAE/L of milk). It is important to notice that gallic, chlorogenic, and ferulic acids were detected in all analyzed milk except in that milk from indoor management. This effect was also observed for catechin, which was not identified in milk from conventional indoor diet. AF pods in addition to indoor diet encouraged the concentration of phenolic compounds and catechin in goat milk. Nevertheless, the greatest value of those bioactive compounds was found in the milk coming from goats in the grazing system. Additionally, milk from animals that grazed/browsed had the best performance (42.1%) to scavenge free radicals in relation to indoor (27.7%) and indoor diet supplemented with AF (30.8%). In addition, a positive correlation was found between the antioxidant activity and the bioactive compound concentrations. In the case of Experiment 5, the total polyphenol content was higher in cheese manufactured with milk from grazing goats (300 mg of GAE/kg of cheese) versus cheese made with milk from goats fed an indoor diet (60 mg of GAE/of cheese). Antioxidant activity followed the same pattern, i.e., cheese from grazing management (24.1%) was superior in comparison to indoor cheese (15.2%). Likewise, caffeic acid was detected exclusively in cheese made with milk from animals under grazing feeding. Interestingly, indoor cheeses reported higher mean values of ferulic acid than grazing cheese (24.1 versus 15.2%). Experiment 3 showed that monoterpene and sesquiterpene contents in goat cheese were superior in grazing management (460 and 850 ng/kg cheese) to indoor feeding (221 and 415 ng/kg cheese), respectively.

In Experiment 6, *Acacia* pods extracts were evaluated for total phenol content (TPC), antioxidant and anti-inflammatory activity in vitro and in vivo as shown in Table 3. TPC ranged from 76 to 620 mg equivalent of gallic acid per gram of extract or 100 g of pods dry matter. In the case of radical scavenging activity of *A. shaffneri* (AS) and *A. farnesiana* (AF) pods extract ranged from 79% to 95%. In the same experiment, with the decolorization reduction of ABTS•^+^ assay, we observed the same performance of the two *Acacia* species (10% of reduction). Lipid peroxidation was inhibited (inhibition of TBARS formation) to the same extent by both *Acacia* species (66%). However, when we evaluated the different extracts, contrasting reduction parameters were observed, ranging from 4% to 17%. AS and AF were able to inhibit the damage induced by reactive oxygen species (ROS). In the same line, oxygen radical absorbance capacity and ferric-reducing antioxidant power showed divergent results, e.g., data registered for ME (450 and 2.0) and MEAE (500 and 1.7). AS and AF extracts evaluated in gerbil plasma proved its positive effects on free radical scavenging (DPPH) and oxygen radical absorbance capacity. In relation to anti-inflammatory activity, AF pod extracts showed important effects on CD-1 mice with induced damage; i.e., all extracts reduced edema and ear thickness induced by TPA (12-O-tetradecanoylphorbol-3-acetate). Additionally, the presence of inflammatory interleukins (Il-1β, Il-6, and TNF-α) was curbed with the use of *Acacia* pod extracts.

### 3.3. In Vivo Prevention of Obesity, Hepatic Steatosis and Insulin Resistance

In Table 4, we describe the results regarding obesity prevention, hepatic steatosis and insulin resistance in mice fed a high-fat diet (HFD) by the intake of goat milk. We observed a reduction on inflammatory markers, an increase of energy expenditure, and higher presence of mitochondrial content in skeletal muscle in mice fed HFD. Our results show that goat milk intake prevents obesity, reduces fat mass, and increases lean mass in the mice fed with HFD. An increase energy expenditure reflected an important intake average of oxygen consumption (VO_2_), which provides an efficient defense against obesity and its related-metabolic diseases as insulin resistance. The latter metabolic abnormality was prevented when the serum insulin in fasting is low, and intraperitoneal glucose tolerance test (ipGTT) reflected a downregulation. Additionally, the size of pancreatic islets was reduced when mice were fed goat milk. HFD feeding caused mitochondria damage function. We also confirmed the ability of goat milk intake to improve mitochondrial function as the results of succinate dehydrogenase (SDH) show activity on the skeletal muscle. Hepatic steatosis appeared as a consequence of overweight. Goat milk effectively prevented hepatic steatosis reducing lipid deposition by quantification of oil red O staining and p-AMPK/AMPK (Phospho-AMPK/AMPK adenine monophosphate (AMP) activated protein kinase) ratio. Goat milk intake protected brown adipose tissue (BAT) from HFD-induced damage. In the same line, BAT expressed UCP-1 protein activity, which plays a key role in the maintenance of body temperature through transforming the energy produced by glucose and fatty acids in mitochondria into heat. Densitometric analysis of phospho-c-Jun N-terminal kinase JNK/JNK ratio in hepatic tissue was reduced by being fed goat milk. Fatty acid bioactive compounds (eicosapentaenoic acid + docosahexaenoic acid)/arachidonic acid ratio) content in hepatic tissue were found in mice fed with goat milk.

## 4. Discussion

### 4.1. Bioactive Compounds in Vegetation Species Browsed/Grazed by Goats on Semiarid Rangelands

As previously mentioned, the present review helps to understand the bioactivity and health properties of goat milk and cheese coming from grazing/browsing management. The screening of vegetation of rangeland plant community, revealed a diverse range of occurrence of plant bioactive compound (PBC) and contrasting amounts. The concentrations varied not only on a plant-to-plant basis, but also among plant portions. Such variation in PBC concentration might bring benefits to animals to better keep the balance among nutritional needs, rumen functioning, and food selection [54,55]. This dissimilarity on PBC is positive since animals can select their diet from a wide range of food choices compared to monoculture pastures where food choices are limited by plant species availability.

The variability of PBC in the animal diet has direct impact on the concentrations of PBC of goat milk (Experiment 2) and cheese (Experiment 5), since a broader variability of phytochemicals was detected in both dairy products from grazing management with respect to indoor management. Therefore, animal production based on grazing/browsing rangeland vegetation seems to be a feasible option for animal keepers not only to feed their animals, but also to obtain further benefits such as bioactivity of animal products [5]. In such conditions animals modulate the ingestion of PBC by shifting the selection from low (e.g., leaves and cladodes) to high PBC (e.g., stems and complete plants) concentrations and vice versa [29]. Therefore, intake choices and animal ingestion behavior have direct impact in terms of bioactivity of milk and cheese [5,10,16,23]. In these farming conditions, the ability of animals to match food items varying in PBC to meet nutritional requirements is remarkable, because it must be underneath the threshold of PBC harmful intake [5,23]. Besides, all plant and plant portions displayed antioxidant activity. Broadly speaking, complete plants yielded the best antioxidant performance, followed by fruits, stems, and leaves. In addition, we identified a positive trend between antioxidant activity versus total polyphenol content (r = 0.948) and antioxidant activity versus flavonoids (r = 0.890). These observations indicate that this outcome could be possible only if other radical scavengers are not present and simultaneously significant contents of TPC and flavonoids are present. This trend can be clearly observed in the pods of *A. farnesiana* which had the largest total polyphenol content (TPC—38,170 mg of GAE/kg DM) among all assessed vegetation which is combined with high antioxidant activity (48%). However, some other feed resources as *A. schaffneri* with lower TPC (2730 mg of GAE/kg DM) had similar antioxidant activity (47%). This indicates that in the latter plant species, there are other bioactive compounds that were not identified in the present study and that are responsible for the displayed antioxidant activity [20]. Therefore, there is a room for improvement in the characterization of bioactive compounds in the rangeland vegetation with significant antioxidant activity.

### 4.2. Bioactive Compounds in Goat’s Milk and Cheese

When we analyzed the antioxidant activity of milk (Experiment 2) and cheese (Experiment 5), a similar pattern as in forages consumed by goats was observed; i.e., antioxidant activity and total phenol content were closely related, where grazing management increased both variables while indoor diets diminished their value. In the same line, the supplementation with a rich source of PBC forage (*A. farnesiana*), increase the occurrence of chlorogenic, ferulic and gallic acids as well as catechin in Experiment 2. In grazing management, not only hydroxycinnamic acids and flavonoids concentration were higher in milk and cheese, but there also are some aromatic components such as monoterpenes and sesquiterpenes that described the same pattern (Experiment 3). However, some of them can be lost upon pasteurization [7]. Indoor feeding instead, increased the concentration of specific PCB as ferulic acid, because maize grain, which is included in the indoor diet formulation, is a rich supply of such phytochemical. Apart from nutritional and hygienic quality, hedonic quality is a major driver of consumers food preference [56], a characteristic that would be improved by the occurrence of PBC in milk and cheese products increasing their acceptance [16]. Because of distinct PBC between milk and cheese, it is expected that consumer preference would also be shaped by this effect. Further investigation on this respect should be done to confirm this hypothesis.

A large body of literature has proved that ruminants fed under grazing/browsing management augment the shares of MUFA and PUFA of milk and cheese while decreasing SFA in comparison to conventional feeding [5,6,7,12,13,29,33]. Though this observation was true for most of the experiments reviewed, we observed inconsistent results. For instance, in Experiment 2, we found that milk from indoor diet showed with the top value of PUFA, MUFA and n-3 fatty acids content. Here, a total mixed ratio with good quality ingredients, including Lucerne hay and grain supplement (60/40%) returned a healthier fatty acid profile. However, conjugated linoleic acid (C18:2 cis-9, trans-11 fatty acid-CLA) in the same experiment was superior in grazing system. The differences could be explained by the distinct biohydrogenation rates as a result of contrasting dietetic ingestion of fatty acids and the dissimilar metabolism of rumen kinetics [1]. In contrast, the value of n-3 fatty acids in Experiments 1 to 5, was numerically superior in grazing systems with respect to indoor diets. Similarly, the n-3/n-6 fatty acid ratio was superior in grazing systems, except in Experiment 3; where indoor diet reported the best value (0.69 vs. 0.60, respectively). Good forage quality of indoor diet, particularly Lucerne hay, could be the factor that promoted higher n-3/n-6 fatty acid ratio. In this respect, Simopoulos [57] demonstrated that human diets with a ratio of n-6:n-3 fatty acids of ~1 promotes better health, whereas higher levels of n-3 fatty acids lowered the incidence of chronic maladies. In the same line, Santurino et al. [24] found that the intake of 60 g/d of goat cheese enriched with n-3 fatty acids and CLA by obese patients, significantly increased plasma high-density lipoprotein (HDL), and apolipoprotein B at the expense of high-sensitivity C-reactive protein, a nitrogenaceous compound related to inflammation and heart disease. In addition, Fontecha and co-authors indicated that the intake of fat from dairy products has no effect on cardiovascular diseases [22]. In that meta-analysis, milk, butter, cheese, yogurt and kefir consumption conversely may have a slight protective effect to the myocardium. PUFA and MUFA of dairy products should extensively contribute to this outcome. Similar results were reported by Intorre et al. [58] who evaluated cheese made from milk produced by cows fed a grass/maize silage supplemented with five percent of linseed oil. In that study, cheese consumption decreased myristic acid (14:0) and low-density lipoprotein (LDL), both metabolites related to atherogenic events. Thus, grazing/browsing management and rich-PUFA diet of ruminants not only exerts healthy benefits of milk and cheese by increasing the n-3 fatty acid concentration, but also do so for human health. In contrast, a recent publication of Jakobsen et al. [59] underlined a direct relationship between intake of high-fat milk and coronary heart disease (CHD) (per 200 g higher intake/day; milk intake range: 0–710 g/day), but no association of low fat milk and CHD was found. Interestingly, the same study reports an inverse relationship between cheese CHD (per 20 g higher intake/day) with a cheese intake range from 0 to 120 g/day. The large heterogeneity of results in that study suggest that some other variables as type of fat or even fatty acid profile should be considered to better interpret the results.

### 4.3. Antioxidant and Anti-Inflammatory Activity of Acacia Pods

Serra and others [16] recently reported that supplementation with polyphenol-rich feeds of animal diets better preserve derived animal products from oxidation and extend the shelf life of food deliveries. In this regard, our group evaluated the transference of bioactivity of two non-conventional natural rich sources of PBC (*A. farnesiana* and *A. schaffneri*) in Experiment 6 and Experiment 7. Both *Acacia* feed resources exhibited significant inhibitory effect on antioxidant capacity. The results reveal that when these rich-PBC forages are included in animal diet, radical scavenger components are transferred firstly from feeds to animal body and metabolism and later to animal products (milk and cheese). Additional benefits of these feeds include the downregulation inflammatory process on ear-edema model in CD-1 mice by *A. farnesiana* induced after TPA (12-O-tetradecanoylphorbol-13-acetate) topical application. The extracts of *A. farnesiana* exhibited significant suppressive effects on interleukins, an inhibitory activity on cyclooxygenase (COX). Likewise, histological analysis demonstrated that pro-inflammatory cytokines as IL-1β, IL-6, IL-10 and TNF-α were inhibited [31]. All these evaluated parameters corroborate the bioactivity effects of *A. farnesiana* to further impact goat metabolism and dairy products bioactivity [15].

### 4.4. In Vivo Prevention of Obesity, Hepatic Steatosis and Insulin Resistance

Anti-obesity, insulin resistance, inflammation, and hepatic steatosis prevention effects of goat milk come from three feeding regimes namely: conventional diet, conventional diet supplemented with 30% *A. farnesiana* and grazing/browsing feeding was assessed. Mice fed high- fat diet supplemented with the tree milk types were able to decrease body weight and body fat mass, improved glucose tolerance, and prevented adipose tissue hypertrophy and hepatic steatosis irrespective of the type of milk. These effects were associated with an increase in energy expenditure, augmented oxidative fibers in skeletal muscle and reduced inflammatory markers [32]. The beneficial effects can be endorsed to polyphenols found in goat milk which augment energy expenditure by modulation of the thermogenic program of subcutaneous adipose tissue and activating AMPK activity in skeletal muscle and hepatic tissue of mice [60]. This effect could be associated to the activation of nuclear receptor PPARγ2 which regulates adipose tissue and the metabolism in skeletal muscle by activating the transcriptional program for mitochondrial biogenesis [61]. Consequently, goat milk can be considered as an alternative to modulate the metabolic alterations induced by high-energetic and high-fat diets. The estimations from the body surface area normalization method yielded a conversion equivalent to daily human intake of 250 mL per glass/day of fresh goat milk for an adult weighing 60 kg to obtain the claimed benefits [32]. This information can have practical implications for future clinical trials aimed to counteract the effects of obesity and related maladies diseases.

## 5. Conclusions

Grazing/browsing is a useful and inexpensive tool to improve the presence of bioactive compounds and thereby increase the bioactivity of milk and cheese. Rangelands concentrate a wide range of plant bioactive compounds. Grazing/browsing on rangelands is superior to indoor feeding to promote the transference of bioactive compounds from vegetation to animal tissues and finally to animal products. In favorable conditions, an indoor diet might be superior to browsing/grazing management in terms of yielding healthier fatty acid profile by increasing PUFA, MUFA, n-3 fatty acids, and ferulic acid content. However, grazing management represents a better option than indoor feeding to enhance bioactivity of milk and cheese. Supplementation with rich-bioactive compounds forages increases total polyphenol, hydroxycinnamic acids, and flavonoid concentrations in milk and cheese. The consumption of goat milk prevents obesity, insulin resistance, inflammation, and hepatic steatosis.

## Figures and Tables

**Table 1:** Antioxidant activity and total polyphenol content of vegetation species browsed/grazed by goats on semiarid rangelands (modified and adapted from Cuchillo et al. 2013 [29]). n = 6.

Specie	Portion	Antioxidant Activity (AA, %)	Total Polyphenol Content (TPC) (mg of Gallic Acid Equivalents/kg DM)
*Aristida adscensionis*	Complete	24.34 ^g^	160 ^l,m^
*Acacia schaffneri*	Complete ^1^	47.38 ^a^	2730 ^b^
*Bouteloua curtipendula*	Complete	35.42 ^d^	314 ^g,h,i^
*Bouteloua repens*	Complete	30.92 ^b,c^	364 ^g^
*Chloris virgata*	Complete	27.39 ^i^	474 ^f^
*Jatropha dioica*	Complete	24.13 ^g^	189l ^k^
*Leptochloa dubia*	Complete	21.48 ^h^	146 ^l,m,n^
*Mimosa biuncifera*	Complete	28.70 ^e,f^	341 ^g,h^
*Rhynchelytrum roseum*	Complete	44.63 ^b,c^	23 ^i,j,k^
*Urochloa fasciculata*	Complete	30.19 ^e^	966 ^c^
Mean value		31.46 ^A^ *	592 ^A^ *
*Acacia farnesiana*	Fruits	47.59 ^a^	38170 ^a^
*Opuntia amyctaea*	Fruits	20.83 ^h^	587 ^e^
*Opuntia hytiacantha*	Fruits	18.30 ^i^	343 ^g,h^
*Prosopis laevigata*	Fruits	22.92 ^g,h^	314 ^g,h^
Mean value		27.41 ^B^ *	9854 ^B^ **
*Celtis pallida*	Leaves	29.49 ^e,f^	280 ^h,i,j^
*Prosopis laevigata*	Leaves	35.68 ^d^	968 ^c^
*Verbesina serrata*	Leaves	17.04 ^i^	272 ^i,j^
Mean value		27.40 ^B^ *	507 ^C^ **
*Celtis pallida*	Stems	44.02 ^c^	370 ^g^
*Verbesina serrata*	Stems	17.78 ^i^	874 ^d^
*Zalazania augusta*	Stems	46.41 ^a,b^	480 ^f^
Mean value		36.07 ^A^ *	575 ^D^ *
*Opuntia affasiacantha*	Cladodes	24.13 ^g^	202 ^k^
*Opuntia hytiacantha*	Cladodes	46.92 ^a^	77 ^n^
*Opuntia robusta*	Cladodes	16.14 ^i^	537 ^e,f^
*Opuntia streptacantha*	Cladodes	16.39 ^i^	147 ^l,m,n^
*Opuntia tomentosa*	Cladodes	16.77 ^i^	335 ^g,h,i^
Mean value		24.07 ^B,C^ **	259 ^E^ *

Complete = mixture of leaves and stems; ^1^ = mix of leaves, stems, and pods. DPPH^+^ = 1, 1-diphenyl-2-picrylhydrazyl (2 mg of DPPH+ in 100 mL of methanol). ^a,b,c,d,e,f,g,h,i,j,k,l,m,n^ Means with different letters within columns are significantly different at alfa= 0.05. Means with capital letters are significantly different (* *p* = 0.05; ** *p* = 0.001) within the column, where: A ≠ B **, A ≠ D *, B ≠ C **, and D ≠ E *.

**Table 2:** Bioactivity and pro-health properties of milk (Experiments 1 and 2) and cheese (Experiments 3, 4, and 5) made with milk coming from goats either grazing/browsing semiarid rangeland or fed indoor diets (modified and adapted from Cuchillo et al., [8]; Cuchillo et al. [7]; Puga et al. [15]; Galina et al. [6] and Puga et al. [13]).

Animal Food Delivery	Milk					Cheese					
	Experiment 1		Experiment 2			Experiment 3		Experiment 4		Experiment 5	
Geographical coordinates	20°35′ Latitude North and 100°18′ Longitude West		20°31′ Latitude North and 100°48′ Longitude West			20°35′ Latitude North and 100°18′ Longitude West					
Dominant vegetation	Shrubby rangeland		Shrubby rangeland			Shrubby rangeland		Shrubby rangeland		Shrubby rangeland	
Altitude a.s.l. (m)	1950		1767			1950		1950		1950	
Treatments	Grazing	Indoor	Grazing	Indoor diet plus pods of *A. farnesiana*	Indoor	Grazing	Indoor	Grazing	Indoor	Grazing	Indoor
Number of animals	10	10	10	10	10	60	60	40	40	10	10
Goat breed	French Alpine	French Alpine	French Alpine	French Alpine	French Alpine	Crossbred French Alpine with Saanen and Toggenburg	Crossbred French Alpine with Saanen and Toggenburg	French Alpine	French Alpine	French Alpine	French Alpine
**Analysis**											
Fatty acids	Gas chromatography		Gas chromatography			Gas chromatography		Gas chromatography		Gas chromatography	
Monoterpenes						Mass spectrometry					
Sesquiterpenes						Mass spectrometry					
Total polyphenols			Folin–Ciocalteu colorimetric method							Folin–Ciocalteu colorimetric method	
Antioxidant activity			DPPH• scavenging activity							DPPH• scavenging activity	
Other bioactive compounds			High-performance liquid chromatography (HPLC)							High-performance liquid chromatography (HPLC)	
**Compounds**	% of total fatty acids										
PUFA	4.73	3.44	4.05	4.74	5.64	6.07	5.24	3.90	5.05	4.80	5.42
MUFA	25.21	19.85	26.22	25.85	31.62	23.49	23.83	22.03	22.08	25.29	23.88
SFA	64.35	70.29	69.73	69.42	62.74	68.88	69.38	57.30	62.21	66.29	65.62
n-3	0.94	0.72	0.87	0.67	0.96	1.27	1.21	1.03	1.0	0.34	0.33
n-3:n-6 ratio	0.35	0.35	0.30	0.21	0.17	0.60	0.69	0.36	0.26	0.36	0.29
CLA (conjugate linoleic acid)	-	-	0.29	0.23	0.20	-	-	-	-	-	-
Monoterpenes (ng/kg of cheese)	-	-	-	-	-	460	221				
Sesquiterpenes (ng/kg of cheese)	-	-	-	-	-	850	415	-	-	-	-
Total polyphenol content (mg of gallic acid equivalents/L of milk or kg of cheese)	-	-	231.6	159.4	305.5	-	-	-	-	300	60
Antioxidant activity (%)	-	-	42.1	30.8	27.7	-	-	-	-	24.1	15.2
Other bioactive compounds	-	-	mg/L							mg/kg	
Chlorogenic acid	-	-	17.64		12.04	-	-	-	-	119	53
Ferulic acid	-	-	6.23	0.0	3.19	-	-	-	-	0.0	165
Gallic acid	-	-	1.43		2.94	-	-	-	-	nd	nd
Catechin	-	-	5.27		1.61	-	-	-	-	0.23	0.16
Quercetin	-	-	nd		nd	-	-	-	-	4.2	nd
Caffeic acid	-	-	nd		nd	-	-	-	-	16	nd

PUFA = polyunsaturated fatty acids; MUFA = monounsaturated fatty acids. SFA = saturated fatty acids. CLA = conjugated linoleic acid isomers (*cis*-9, *trans-*11; *trans-*9, *cis-*11; *trans-*10, *cis-*12, *cis-*10, *cis-*12). DPPH• = 2,2-diphenyl-1-picrylhydrazyl; nd = not detected.3.3. Antioxidant and anti-inflammatory activity of acacia pods extracts.

**Table 3:** Goat feeds as bioactive compounds sources: in vitro and in vivo effects on bioactivity and pro-health properties (modified and adapted from and Puga et al. [30] and Delgadillo et al. [31]).

	Experiment 6		Experiment 7					
			In Vitro					
Variables	AS	AF	*Acacia farnesiana* Pods Extracts					
			CE	HE	KE	ME	MEAE	AE
Total polyphenol (TP) mg of equivalent of gallic acid/g of extract	213 ^a^	76 ^b^	TPC, mg of equivalent of gallic acid/100 g pods, dry matter					
			506 ^c^	620 ^a^	594 ^b^	378 ^d^	399 ^d^	565 ^b^
Free radical scavenging capacity % (DPPH^+^)	95 ^a^	95 ^a^	90 ^a^	80 ^b^	79 ^b^	82 ^b^	80 ^b^	80 ^b^
Free radical scavenging capacity % (ABTS•^+^)	10 ^a^	10 ^a^	-	-	-	-	-	-
Inhibition of TBARS formation, %	66 ^a^	66 ^a^	IC_50_ on TBARS ug/mL					
			9 ^b^	12 ^a^	4.0 ^c^	4.5 ^c^	4.5 ^c^	17 ^a^
Protection against H_2_O_2_ oxidative-induced damage ^†^; (%)	++	+++	37 ^a^	25 ^b^	36 ^a^	18 ^c^	23 ^b^	18 ^c^
Oxygen radical absorbance capacity, (ORAC) ^§^	-	-	150 ^c^	100 ^d^	450 ^b^	450 ^b^	500 ^a^	50 ^e^
Ferric-reducing antioxidant power (FRAP) ^¥^	-	-	1.5 ^a^	1.4 ^a^	1.5 ^a^	2.0 ^a^	1.7 ^a^	1.4 ^a^
			**In Vivo**					
Free radical scavenging capacity, % (DPPH^+^)	29	30	-	-	-	-	-	-
Oxygen radical absorbance capacity (ORAC)≠	2207	1561	-	-	-	-	-	-
Edema (mg)	-	-	12 ^a^	13 ^a^	13 ^a^	11 ^a^	14 ^a^	14 ^a^
MPO inhibition %	-	-	34.9 ^b^	28.5 ^b^	65.9 ^a^	31.0 ^b^	73.3 ^a^	37.1 ^b^
Interleukins IL-1β (pg/biopsy) ^Š^	-	-	52 ^b^	60 ^a^	55 ^b^	52 ^b^	50 ^b^	52 ^b^
Interleukins IL-6 (pg/biopsy) ^Š^	-	-	800 ^b^	1200 ^a^	780 ^b^	1100 ^a^	1200 ^a^	1100 ^a^
TNF-α ^Š^	-	-	15 ^b^	12 ^b^	17 ^a^	13 ^b^	22 ^a^	11 ^b^
Ear thickness (µm)	-	-	400 ^b^	420 ^b^	580 ^a^	580 ^a^	300 ^c^	400 ^b^
Prostaglandin (µg/mL)	-	-	1200 ^b^	900 ^d^	3000 ^c^	16,000 ^a^	11,000 ^b^	12,000 ^b^

AS = *Acacia schaffneri*; AF = *Acacia farnesiana*; DPPH^+^ = 1,1-diphenyl-2-picrylhydrazyl; ABTS = 2,2′-azino-bis (3-ethylbenzthiazoline-6-sulphonic acid); TBARS = thiobarbituric acid reactive substances; ORAC = oxygen radical absorbance capacity; FRAP = ferric reducing antioxidant power. ^†^ Extract crude added at 200 ppm. ^§^ μm of Trolox equivalents/g of extract. ^§^ mmol of Trolox equivalents/mL of extract. ≠ Oxygen radical absorbance capacity (ORAC) (μM Trolox equivalents/L) of gerbil (*Meriones unguiculatus*) plasma. MPO = oxidative enzyme myeloperoxidase. OD = optic density. Interleukins IL-1ß (pg/biopsy). ^Š^ Interleukin in supernatants of homogenates from CD-1 mice ears after treatment with different extracts from AF pods. CE = chloroformic extract. HE = hexanic extract. KE = ketonic extract. ME = methanolic extract. MEAE = methanolic:aqueous extract. AE = aqueous extract. CD = goat’s milk from conventional diet. G = goat’s milk from grazing diet. CD + AF = goat’s milk from conventional diet + 30% of *Acacia farnesiana* pods flour diet. ^a,b,c,d,e^ Means with different letters within rows are significantly different at α = 0.05.

**Table 4:** Goat milk used as bioactive compounds source: in vivo effects on bioactivity and pro-healthy properties against induced obesity (modified and adapted from Delgadillo–Puga et al. [32]).

	Experiment 8				
Variables	Control	HFD	CD	G	CD + AF
	Energy intake and body changes				
Final body weight (g)	30 ^b^	42 ^a^	29.2 ^b^	34.5 ^b^	33.8 ^b^
Body weight gain (g)	9.5 ^c^	21. 6 ^a^	9.6 ^c^	16.0 ^a,b^	11.8 ^b,c^
Food intake (g/day)	3.2 ^a,b^	3 ^d^	3.7 ^c^	4.4 ^a^	3.9 ^b^
Energy intake (kcal/day)	16.1 ^c^	16 ^c^	18.5 ^b^	21.7 ^a^	18.9 ^b^
Mice body composition Fat mass (%)	20 ^b^	35 ^a^	10.5 ^c^	19.5 ^b,c^	19.1 ^b^
Mice body composition Lean mass (%)	75 ^b^	62 ^c^	86.0 ^a^	78.5 ^a,b^	77.7 ^d^
	Serum and insulin resistance related-parameters				
Serum glucose (mg/dL)	200 ^b^	310 ^a^	228 ^b^	263 ^a,b^	242 ^a,b^
Serum insulin (ng/mL)+	1 ^a.b^	2.8 ^a^	0.63 ^b^	0.65 ^b^	0.70 ^b^
ipGTT AUC	25,000 ^b^	45,000 ^a^	19,584 ^b^	28,415 ^b^	22,689 ^b^
ipITT AUC	13,240 ^a,b^	20,000 ^a^	14,599 ^a,b^	13,240 ^a,b^	1741 ^b^
Pancreatic islets size (µm^2^)	9800^b^	20,000 ^a^	8508 ^b^	10,110 ^a,b^	6683 ^b^
	Energy expenditure and thermogenic changes				
Average VO_2_ (oxygen consumption; mL/kg/h) feeding	5700 ^b^	5450 ^c^	6035.1 ^a^	6059 ^a^	5989 ^a^
Lipid content BODIPY staining in skeletal muscle (relative units)	50 ^b^	150 ^a^	38.5 ^b^	24.33 ^b^	16.57 ^b^
Mitochondrial activity SDH staining in skeletal muscle (relative units)	1900 ^b^	900 ^c^	2237 ^a,b^	2612 ^a^	3075.0 ^a^
UCP-1/GAPDH immunoblotting BAT (relative units)	1 ^c^	1.5 ^b^	1.5 ^b^	2.0 ^a^	2.0 ^a^
	Hepatic energy improvement and anti-inflammatory prevention				
Lipid content ORO staining in hepatic tissue (relative units)	105.8 ^b^	300 ^a^	105 ^b^	93.48 ^b^	104.4 ^b^
p-AMPK/AMPK ratio in hepatic tissue (relative units) by immunoblotting	1 ^c^	1.0 ^c^	1.41 ^b,c^	1.82 ^b^	2.59 ^a^
p-JNK/JNK ration in hepatic tissue (relative units) by immunoblotting	1 ^a^	3.00 ^a^	0.85 ^a^	0.37 ^b^	0.56 ^a^
(EPA+DHA)/AA ratio in liver (mg/g of liver)	0.2 ^c^	0.2 ^c^	1.39 ^b,c^	2.07 ^a,b^	0.82 ^b,c^

AS = *Acacia schaffneri*; AF = *Acacia farnesiana*; HFD = High fat diet without goat’s milk. CD = goat’s milk from conventional diet. G = goat’s milk from grazing diet. CD + AF = goat’s milk from conventional diet + 30% of *A. farnesiana* pods meal diet. ipGTT AUC = intraperitoneal glucose tolerance test area undercurve. ipITT AUC = intraperitoneal insulin tolerance test area undercurve. SDH = densitometric quantification of muscle succinate dehydrogenase. ORO = densitometric quantification of Oil red O staining. UCP-1 = uncoupling protein one. BAT = brown adipose tissue. p-AMPK/AMPK = densitometric analysis of phospho-AMPK/AMPK adenine monophosphate (AMP) activated protein kinase ratio. p-JNK/JNK = densitometric analysis of phospho-c-Jun N-terminal kinase JNK/JNK ratio. (EPA+DHA)/AA = (eicosapentaenoic acid + docosahexaenoic acid)/arachidonic acid ratio. Results are presented as the mean, *n* = 6 mice per group and evaluated by one-way ANOVA followed by Tukey multiple comparison post hoc test. The differences were considered statistically significant at *p* < 0.05. Mean values with different lowercase letters show statistical differences among them. ^a,b,c,d^ Means with different letters within rows are significantly different at α = 0.05.

## Data Availability

Not applicable.
